# Replication-biased genome organisation in the crenarchaeon *Sulfolobus*

**DOI:** 10.1186/1471-2164-11-454

**Published:** 2010-07-28

**Authors:** Anders F Andersson, Erik A Pelve, Stefan Lindeberg, Magnus Lundgren, Peter Nilsson, Rolf Bernander

**Affiliations:** 1 Department of Ecology and Evolution, Evolutionary Biology Centre, Uppsala University, Uppsala, Sweden; 2 Science for Life Laboratory, KTH Royal Institute of Technology, Stockholm, Sweden; 3 Department of Molecular Evolution, Evolutionary Biology Centre, Uppsala University, Uppsala, Sweden; 4 Department of Cell and Molecular Biology, Biomedical Centre, Uppsala University, Uppsala, Sweden; 5 Department of Proteomics, School of Biotechnology, KTH Royal Institute of Technology, Albanova University Centre, Stockholm, Sweden

## Abstract

**Background:**

Species of the crenarchaeon *Sulfolobus *harbour three replication origins in their single circular chromosome that are synchronously initiated during replication.

**Results:**

We demonstrate that global gene expression in two *Sulfolobus *species is highly biased, such that early replicating genome regions are more highly expressed at all three origins. The bias by far exceeds what would be anticipated by gene dosage effects alone. In addition, early replicating regions are denser in archaeal core genes (enriched in essential functions), display lower intergenic distances, and are devoid of mobile genetic elements.

**Conclusion:**

The strong replication-biased structuring of the *Sulfolobus *chromosome implies that the multiple replication origins serve purposes other than simply shortening the time required for replication. The higher-level chromosomal organisation could be of importance for minimizing the impact of DNA damage, and may also be linked to transcriptional regulation.

## Background

Genes are non-randomly ordered on chromosomes. In eukaryotes, co-expressed genes tend to cluster across all kingdoms [1-4], with cluster sizes ranging from kilobases in yeast to megabases in mammals [5]. Co-expression of neighbouring genes may result from use of common promoters or upstream activating sequences, while the transcriptional activity of larger chromosome domains is regulated by the structure of the chromatin and/or its spatial positioning within the nucleus [6]. Some of the observed grouping of co-expressed genes likely reflects clustering of functionally related genes [7], while some may be attributed to transcriptional leakage [8].

In bacteria and archaea, the most obvious case of gene clustering is the organisation of genes into co-transcribed cassettes, operons. This facilitates tight co-regulation of genes encoding proteins involved in the same cellular pathway, or of subunits of the same protein complex [9,10]. Adjacent location of multi-gene functional entities also increases the chance for their co-transfer in lateral transfer events, likely to be important for efficient horizontal propagation [11,12]. Conserved bidirectionally transcribed gene pairs have also been observed, typically involving a transcriptional regulator that shares the promoter region with a target operon [13]. Non-random gene order is also evident at much larger scales [14], such as a 600 - 700 kb periodic pattern of gene co-expression observed in *Escherichia coli *and *Bacillus subtilis *[15,16], which likely reflects how the chromosome is spatially structured in the nucleoid. In bacteria, other trends have also been observed, such as higher incidence of essential genes on the leading strand [17], clustering of evolutionary persistent genes [12], and clustering of genes involved in transcription and translation near the origin of replication in fast-growing bacteria [18].

Archaeal organisms exhibit both bacterial and eukaryotic-like features. In particular, the information-processing systems (replication, transcription, translation) closely resemble their eukaryotic counterparts [19]. *Sulfolobus *species are thermoacidophilic crenarchaea, serving as model systems for the archaeal cell cycle [20]. The *Sulfolobus *cell cycle is characterised by a short pre-replicative phase, an S phase of about a third of the generation time, and a long post-replicative phase [21,22]. Global gene expression analysis has revealed that at least 10% of the *Sulfolobus acidocaldarius *genes display cyclic expression during cell cycle progression [23], including a unique cell division machinery, the Cdv system, that recently was identified based on the expression data [24,25].

**Figure 1:**
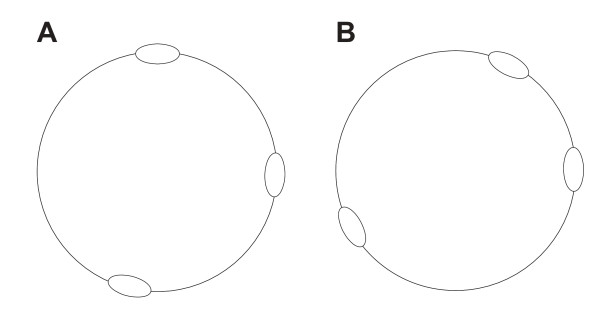
**Schematic representation of the *S. acidocaldarius *(A) and *S. solfataricus *(B) chromosomes**. The ellipses indicate replication origins (positions from [26]).

In contrast to all studied bacteria and most archaea, *Sulfolobus *chromosomes harbour multiple origins of replication. Marker frequency analysis has shown that replication is initiated in near synchrony at the three origins and, due to the uneven spacing of the origins, asynchronously terminated on the circular chromosome [26]. The selective advantage of multiple origins (if any) is not clear: the fact that the origins are unevenly distributed on the chromosome (Figure 1) is, for instance, not in agreement with models in which shortening of the replication time would be the main selective force.

Here, we performed global gene expression analysis in exponentially and stationary phase cells, and investigated other properties of genome organisation in *Sulfolobus*. The results demonstrate that the *Sulfolobus *chromosome is organised in a highly replication-biased manner, such that levels of gene expression, as well as genome sequence-derived parameters, are correlated with distance to nearest replication origin.

## Results

To monitor the distribution of gene expression over the *Sulfolobus *chromosome, we harvested RNA from *Sulfolobus solfataricus *and *S. acidocaldarius *cell cultures in exponential and stationary phase. The RNA was reverse transcribed into cDNA, labelled and hybridised onto spotted whole-genome DNA microarrays [27]. To compensate for differences in array probe concentrations and hybridisation efficiencies, the cDNA was co-hybridised with differentially labelled genomic DNA derived from stationary phase cultures. As stationary phase cells exclusively contain fully replicated chromosomes [21] and all genes, consequently, are present in equal copy number, the cDNA/genomic DNA ratios serve as estimates of relative transcript abundances.

**Figure 2:**
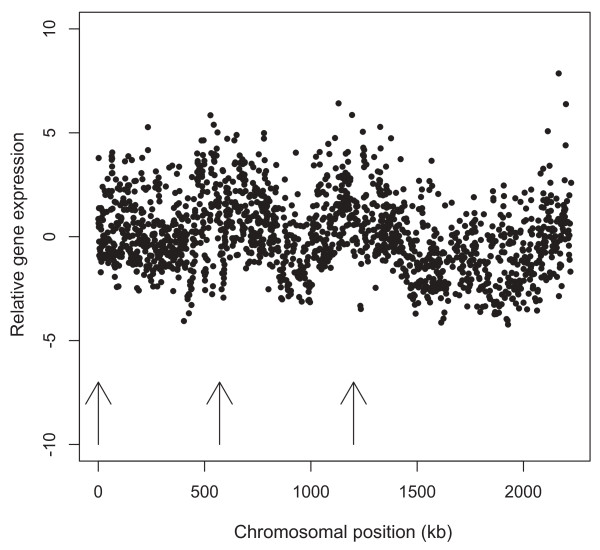
**Distribution of gene expression over the *S. acidocaldarius *chromosome in exponentially growing cultures**. Each filled circle represents a single gene, with expression provided as log_2_-transformed (cDNA/genomic DNA) ratio. Arrows indicate positions of replication origins.

**Figure 3:**
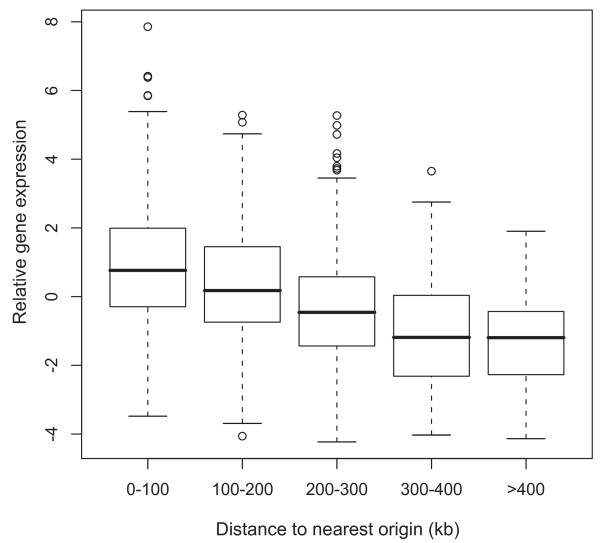
**Distribution of expression for genes within indicated intervals of distance to nearest replication origin in exponential phase cultures of *S. acidocaldarius***. Fifty percent of the data points reside within boxes, 75% within whiskers, and medians are indicated by horizontal lines within boxes (open circles indicate individual genes). Gene expression was significantly negatively correlated with distance to nearest origin (Spearman rank-order correlation, *ρ *= -0.41; *P *< 10^-15 ^calculated on individual genes).

Gene expression was non-randomly distributed over the chromosomes in both species (Figure 2; Additional file 1, Figure S1). Transcriptionally active regions coincided with replication origins, and gene expression was negatively correlated with distance to the nearest origin in both genomes (Figure 3; Additional file 1, Figure S2). The gene expression gradients were significantly more pronounced than what would be anticipated from gene dosage effects alone in growing populations. Thus, while the average gene copy number ratio between the earliest and latest replicating chromosome regions in a growing *Sulfolobus *population is about 1.3 fold [26], the average expression ratio between genes located proximally and distantly relative to the nearest origin was >4 fold (Figure 3). A negative, but weaker, correlation to distance from the nearest origin was observed also when cDNA derived from *S. acidocaldarius *cells in stationary phase was hybridised (Additional file 1, Figure S3), whereas in *S. solfataricus *there was no such correlation (data not shown).

**Figure 4:**
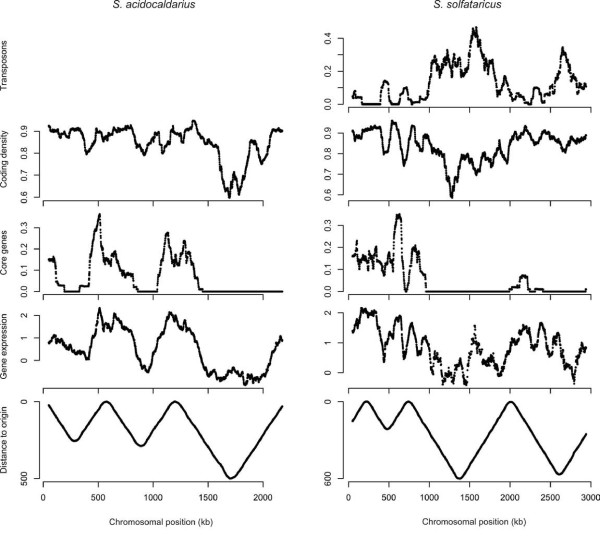
**Distance to nearest origin, average gene expression, proportion of archaeal core genes, protein-coding density, and transposable element density over the chromosome of *S. acidocaldarius *(left panel) and *S. solfataricus *(right panel) in 100 kb sliding windows, translocated in 1 kb steps**. Chromosomal positions for window mid-points are shown on the x-axis.

Comparative genomics has revealed a core of 166 genes present in all archaea [28]. Evolutionary persistent genes like these are highly enriched in house-keeping functions essential to the organism [29], and such genes have been found to cluster in both bacteria [12] and eukaryotes [2]. We found that genes representing clusters of orthologous groups (COGs; [30]) present in all archaea (archaeal core genes) clustered in the early replicating regions and were absent in large regions of the genomes (Figure 4), and that the density of archaeal core genes was significantly negatively correlated with distance to nearest origin in both organisms (Table 1). Also ribosomal RNA (rRNA) genes and transfer RNA (tRNA) genes clustered in the highly expressed regions (Additional file 1, Figure S4).

**Table 1:** Correlations between distance to nearest origin, average gene expression in exponential phase, proportion archaeal core genes, protein-coding density, average distance between adjacent bidirectional (divergently transcribed) genes, and transposable element density in the two *Sulfolobus *chromosomes

	Gene expression	Archaeal core genes	Coding density	Bidirectional gene pair distances	Proportion transposons
	*S. solfataricus*				
Distance to origin	-0.68 (10^-3^)	-0.62 (10^-2^)	-0.64 (10^-2^)	0.57 (10^-2^)	0.71 (10^-3^)
Gene expression		0.83 (10^-5^)	0.72 (10^-3^)	-0.71 (10^-3^)	-0.64 (10^-2^)
Archaeal core genes			0.74 (10^-4^)	-0.58 (10^-2^)	-0.75 (10^-4^)
Coding density				-0.92 (10^-5^)	-0.87 (10^-6^)
Bidirectional gene distances					0.74 (10^-4^)
	*S. acidocaldarius*				
Distance to origin	-0.83 (10^-5^)	-0.77 (10^-4^)	-0.64 (10^-2^)	0.57 (10^-2^)	
Gene expression		0.85 (10^-6^)	0.70 (10^-3^)	-0.67 (10^-3^)	
Archaeal core genes			0.51 (10^-1^)	-0.59 (10^-2^)	
Coding density				-0.79 (10^-5^)	
Bidirectional gene distances					

The genomic parameters were calculated in 100 kb, non-overlapping windows (22 and 29 windows for *S. acidocaldarius *and *S. solfataricus*, respectively). Numbers indicate Spearman rank-order correlation coefficients, *ρ*, with *P *values (rounded upwards) within parenthesis.

Compared to eukaryotes, prokaryotic genomes are highly compact, with short intergenic regions and generally few repeated elements. Nevertheless, elevated gene densities were observed in the early replicating (highly expressed) regions (Figure 4). Gene density was hence negatively correlated with distance to nearest origin, and positively correlated with gene expression, in both genomes (Table 1). As this could potentially be a consequence of that highly expressed genes relatively frequently belong to polycistronic transcripts, with short intergenic regions, we investigated distances between adjacent genes on opposite strands (thus not belonging to the same transcript). These distances increased with distance to origin (Table 1), indicating that the increased gene density in early replicating regions could not be explained only by an increased operon incidence.

The *S. solfataricus *chromosome is one of the most transposon-dense of all sequenced genomes [31]. Also for this feature a non-random distribution could be observed, as also noted previously [32], with transposon density being positively correlated with distance to nearest origin (Figure 4; Table 1).

## Discussion

We observed a strongly replication-biased genome organization in the two *Sulfolobus *species, despite that massive genomic rearrangements have occurred since the organisms diverged ([32]; Additional file 1, Figure S5), which indicates that the trait is under selection. Replication-biased genome organisation has also been reported in bacteria (reviewed by [33]). The nature of the *Sulfolobus *genome organisation is, however, different in several aspects. Fast-growing bacteria with multiple simultaneously ongoing rounds of replication (and hence high origin-to-terminus ratios) display increased densities of highly expressed genes (genes with high codon adaptation index) near origins. However, the relationship only holds true for genes involved in transcription and translation and has been suggested to reflect selection for gene dosage effects, advantageous during rapid growth [18]. In contrast, in *Sulfolobus *the correlation between gene expression and distance to origin remains even if transcription and translation genes are excluded (Spearman *P *< 10^-12 ^for both genomes). Moreover, in slow-growing bacteria (comparable to *Sulfolobus *with 6 - 8 hour doubling time) only rRNA genes are generally located close to origins [18].

If the replication-biased genome organisation in *Sulfolobus *does not correspond to selection for gene dosage effects, what does it reflect? The clustering of archaeal core genes near the origins may indicate selection for early replication of essential genes, since evolutionary persistent genes often are essential [29]. Also, since essential genes often are highly expressed (core genes are significantly higher expressed than non-core genes; Mann-Whitney *P *= 0.029 in *S. acidocaldarius*), the observed expression gradient could be a secondary effect of their biased distribution. To evaluate if core genes cluster near origins independently of expression level, we binned the *S. acidocaldarius *genes based on expression levels into 12 equally sized bins (Additional file 1, Figure S6). The binning was sufficiently fine-grained to remove expression differences between core and non-core genes; within the 11 bins that included genes of both categories there were no significant differences in expression levels between the two groups (Mann-Whitney *P *> 0.23 for all bins). However, the distance to nearest origin was significantly shorter (Mann-Whitney *P *< 0.05) for core than non-core genes in 8 out of 11 bins, showing that essential genes cluster near origins independently of expression level, and may, thus, cause the correlation between expression and distance to origin. If so, one would perhaps expect the expression gradient to disappear when analysing core and non-core genes separately. This is however not the case; although weaker than for the whole dataset, the correlations remain for both groups of genes (Spearman *ρ *= -0.19, *P *< 0.05 and *ρ *= -0.39, *P *< 10^-15 ^for core and non-core genes, respectively). However, the extent to which individual genes contribute to fitness likely varies within the two groups, and a more fine-grained binning according to fitness contribution would be needed to determine its effect on the expression gradient.

The multiple origins may hence potentially serve to promote fast backup of essential genetic material. This could also explain the shorter intergenic distances, lack of transposons, and clustering of rRNA and tRNA genes in early replicating regions. Having two copies of a gene facilitates expression of a functional protein even if one copy is damaged, thereby preventing cell death. The intact chromosomal copy could potentially also facilitate repair of the damaged DNA by homologous recombination. In support, sister chromatid junctions have been observed near replication origins in *S. solfataricus *[34].

*Sulfolobus *species inhabit geothermal environments where thermal DNA damage, in particular deamination, depurination and oxidation, are prone to occur frequently [35]. In addition, the aerobic metabolism requires surface growth with consequent UV exposure. It has been demonstrated that the DNA repair systems of *Sulfolobus *are constitutively expressed in batch cultures [36,37], reflecting this life style, and that expression is correlated to the replicative cell cycle stage [23]. A need for backup of genetic material would also be in line with the organisation of the *Sulfolobus *cell cycle, in which replication is initiated shortly after cell division, and two complete chromosomes thus are present during most of the cell cycle, as well as in all cells in stationary phase [21]. This organisation of the cell cycle has been shown to be widely conserved among crenarchaea [38]. However, despite the fact that pronounced clustering of archaeal core genes, coinciding with elevated coding densities, is apparent in all Sulfolobales genomes (including *Metallosphaera sedula*), this does not appear to be a general feature of crenarchaea (Additional file 1, Figure S4). It is possible that the anaerobic lifestyle characteristic of most other genome-sequenced crenarchaea may reduce the mutation frequency and, consequently, the selective advantage of this higher-order genome structure.

Our data indicate that genome organisation reflects selection for early backup of essential genetic material, but we cannot rule out that other selective forces may affect gene localisation. In higher eukaryotes, chromosome structure is tightly linked to gene regulation and to replication timing [39]. Although a suite of architectural proteins have been identified and characterised in archaea [40], relatively little is known about archaeal chromosome structure and its potential role in gene regulation [41]. However, the identification of DNA-binding proteins that can undergo methylation [42] and acetylation [43], as well as expression of chromatin-organizing proteins that is dependent on growth phase [44] and cell cycle progression [23], indicate dynamic chromosome structures in archaea which may have implications for transcriptional regulation. Thus, the organisation of highly expressed genes near replication origins in *Sulfolobus *might, in addition to selective forces related to DNA repair and genetic back-up, reflect a higher order chromosome structure centred at replication origins.

A recent comparative genomics study of seven *S. islandicus *isolates revealed a large genomic region that was enriched in gene insertion and deletions [45]. Our analysis shows that this region coincides with a region that is very low in archaeal core genes and has low gene density (Additional file 1, Figure S4). Whether this reflects selection against insertions and deletions in regions dense in essential genetic material, or that the chromosome structure of these regions physically prevents recombination events, is an interesting topic for further investigation.

## Conclusion

Our study reports a strong replication-biased structuring of the *Sulfolobus *chromosome which implies that the multiple replication origins serve purposes other than only shortening the time required for replication. The higher-level chromosomal organisation may be of importance for minimizing the impact of DNA damage during growth in extreme environments and is possibly related to chromosome structure. The findings provide a basis for further investigation of chromosome organisation, transcription patterns and gene regulation in archaea, as well as of the evolutionary forces that promote different levels of transcriptional and chromatin organisation.

## Methods

### Cell cultivation

*S. acidocaldarius *DSM 639 and *S. solfataricus *DSM 1617 cultures were grown at 79°C in modified Allen [46] mineral base medium containing 0.2% tryptone. Growth was monitored by optical density (OD) measurements at 600 nm. Samples for RNA preparation were extracted from exponentially growing cultures at OD 0.1, and for RNA and DNA preparation from stationary phase at OD 0.6 for *S. acidocaldarius *and at OD 0.7 for *S. solfataricus*. DNA content and cell size distributions were analysed by flow cytometry as described [21], to confirm that the cells were in exponential and stationary phase, respectively (data not shown).

### Extraction of RNA and DNA

RNA was extracted as described in the protocol "RNA Extraction from Sulfur-utilizing Thermophilic Archaea" protocol in the Archaea manual [47] with an additional DNase I treatment and phenol purification step. DNA was extracted as described previously [26].

### Labelling of cDNA and genomic DNA

Five μg of total RNA was reverse transcribed into Cy5/Cy3-labelled cDNA using aminoallyl-modified nucleotides, as described http://www.biotech.kth.se/molbio/microarray/. Stationary phase genomic DNA (1.4 μg) was labelled with Cy3/Cy5-dUTPs, as described [26].

### Microarray analysis of transcript abundance

Microarrays with gene-specific tags (GSTs) were produced as previously described [27]. Probes were printed in duplicates on Ultra GAPS slides (Corning) at the KTH Microarray Center. cDNA from exponential and stationary phase cultures was co-hybridised in triplicates with genomic DNA from stationary phase cells for 16 - 20 hr as described http://www.biotech.kth.se/molbio/microarray/. After washing, slides were scanned with an Agilent Scanner (Agilent Technologies) and data was collected with GenePix 5.0 software (Axon Instruments). Low-quality spots were excluded as described [48]; 1288 and 1210 *S. solfataricus *and 1650 and 1667 *S. acidocaldarius *genes remained after filtering for exponentially and stationary phase, respectively. Cy5/Cy3 log_2 _ratios of background-subtracted intensities were extracted, and each array was normalised such that the mean log ratio equalled zero. For each gene the log ratio was averaged first over probe replicates and then over arrays. The microarray data have been deposited in ArrayExpress (E-MEXP-2770).

### Genomic analysis

Genomic data on sequenced archaeal genomes was downloaded from National Center for Biotechnology Information ftp://ftp.ncbi.nlm.nih.gov/genomes/Bacteria/ in December 2008 and was later supplemented with data on two *S. islandicus *genomes. Orthologous gene pairs for *S. acidocaldarius *and *S. solfataricus *were identified with Inparanoid [49]. Archaeal core genes were defined as COGs present in all completed archaeal genomes, excluding the symbiont *Nanoarchaeum equitans*. Since the aim of this was to identify essential genes, in each genome COGs that were represented by multiple genes were excluded from the core (for instance COG0183 (acetyl-CoA acetyltransferase) with 11 copies in *S. acidocaldarius*), since not all of these proteins were likely to be essential. Data plotting and statistical analysis was performed in *R *http://www.r-project.org.

## Competing interests

The authors declare that they have no competing interests.

## Authors' contributions

AFA and RB designed the study. AFA and EAP analysed the data. EAP, SL and ML conducted the experiments. All authors wrote the paper and read and approved the final manuscript.

## Supplementary Material

Additional file 1 **Supplementary figures**. Supplementary figures 1-6. Click here for file
