# Supplementary material for: Replication-biased genome organisation in the crenarchaeon Sulfolobus
Source: BMC Genomics. 2010 Jul 28;11:454. doi: 10.1186/1471-2164-11-454 (PMC3091651; doi:10.1186/1471-2164-11-454)
Supplement: Additional file 1 — Supplementary figures. Supplementary figures 1-6. [file 1471-2164-11-454-S1.DOC]

**
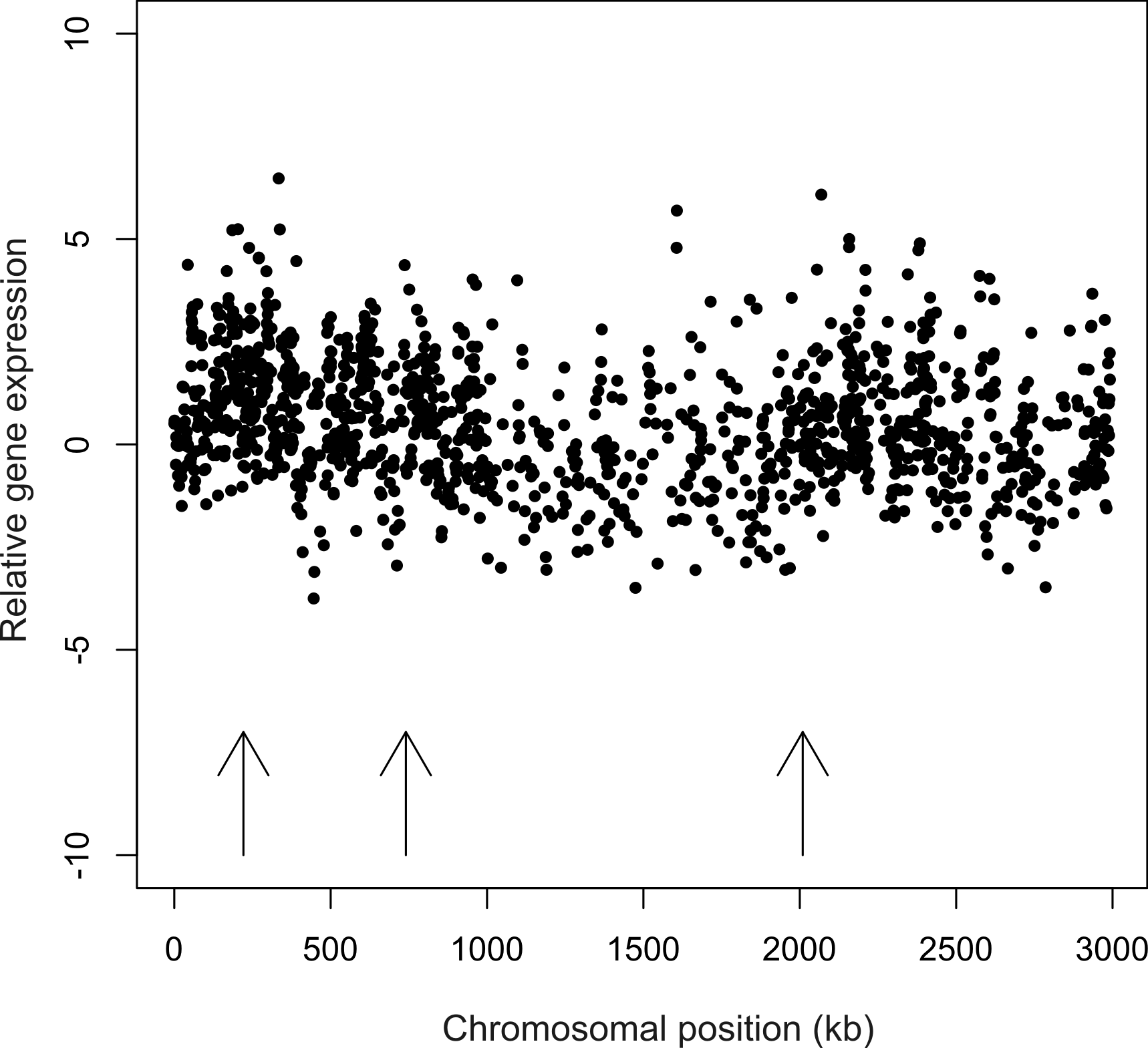
**

**Supplementary Figure 1.** Distribution of gene expression over the *S. solfataricus* chromosome in exponentially growing cultures. Each filled circle represents a single gene, with expression provided as log_2_-transformed (cDNA/genomic DNA) ratio. Arrows indicate positions of replication origins.


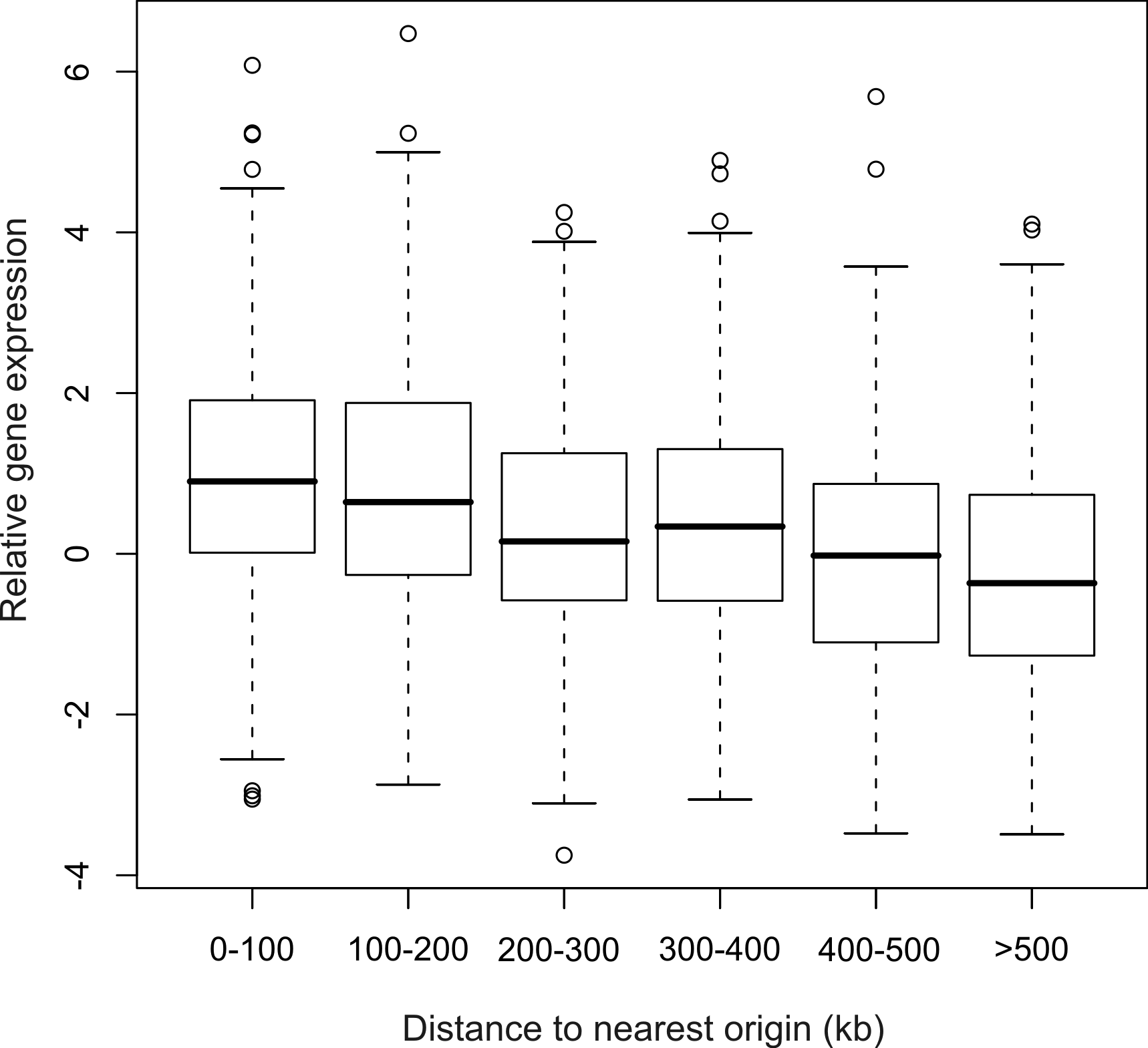


**Supplementary Figure 2.** Distribution of gene expression for genes within indicated intervals of distance to nearest replication origin in exponential phase cultures of *S. solfataricus.* Fifty percent of the data points reside within boxes, 75% within whiskers, and medians are indicated by horizontal lines within boxes (circles indicate individual genes). Gene expression was significantly negatively correlated with distance to nearest origin (Spearman rank-order correlation, *ρ* = -0.26*, P* <10^-15^).


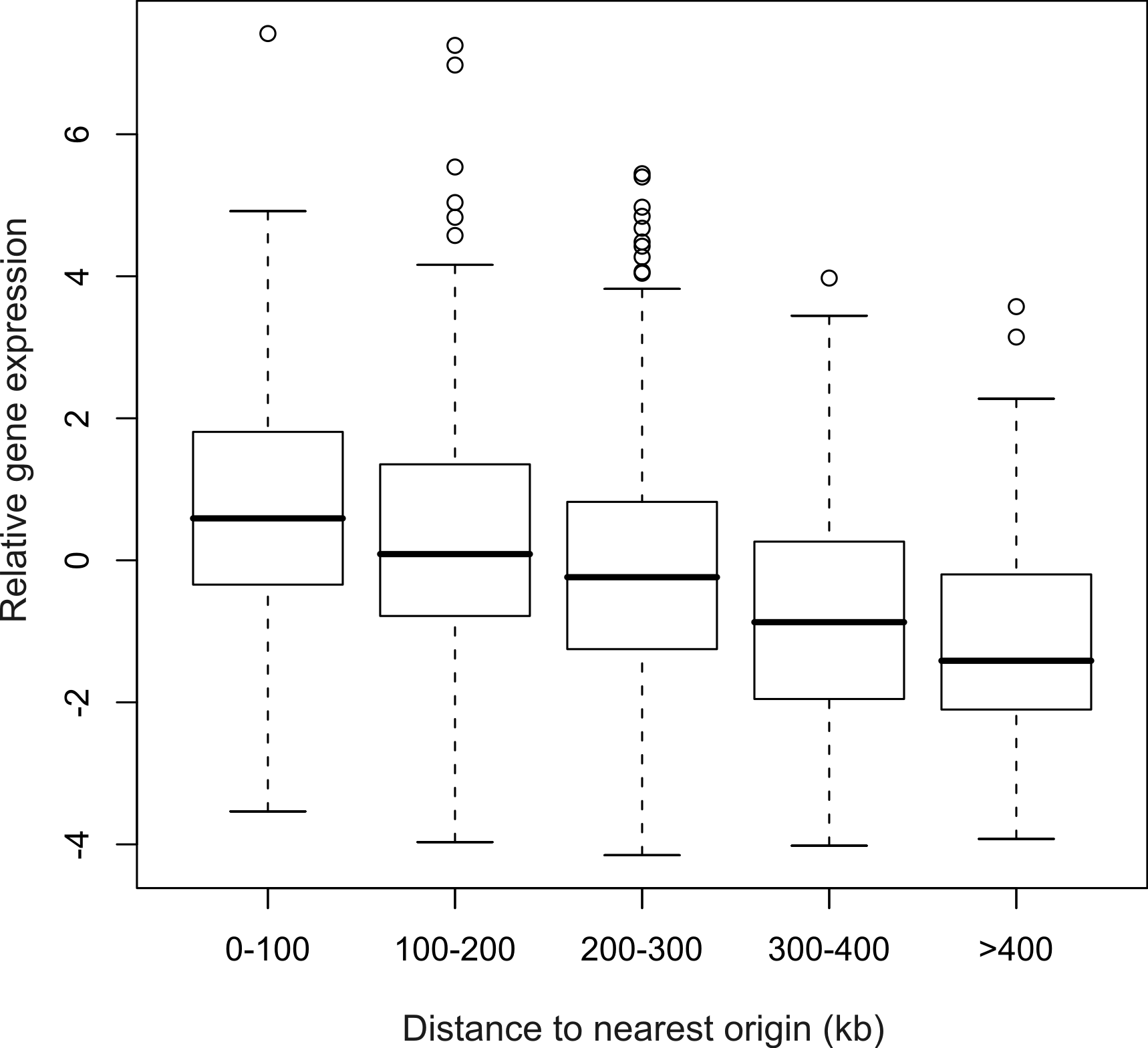


**Supplementary Figure 3.** Distribution of gene expression for genes within indicated intervals of distance to nearest replication origin in stationary phase cultures of *S. acidocaldarius.* Fifty percent of the data points reside within boxes, 75% within whiskers, and medians are indicated by horizontal lines within boxes (circles indicate individual genes). Gene expression was significantly negatively correlated with distance to nearest origin (Spearman rank-order correlation, *ρ* = -0.32*, P* <10^-15^).


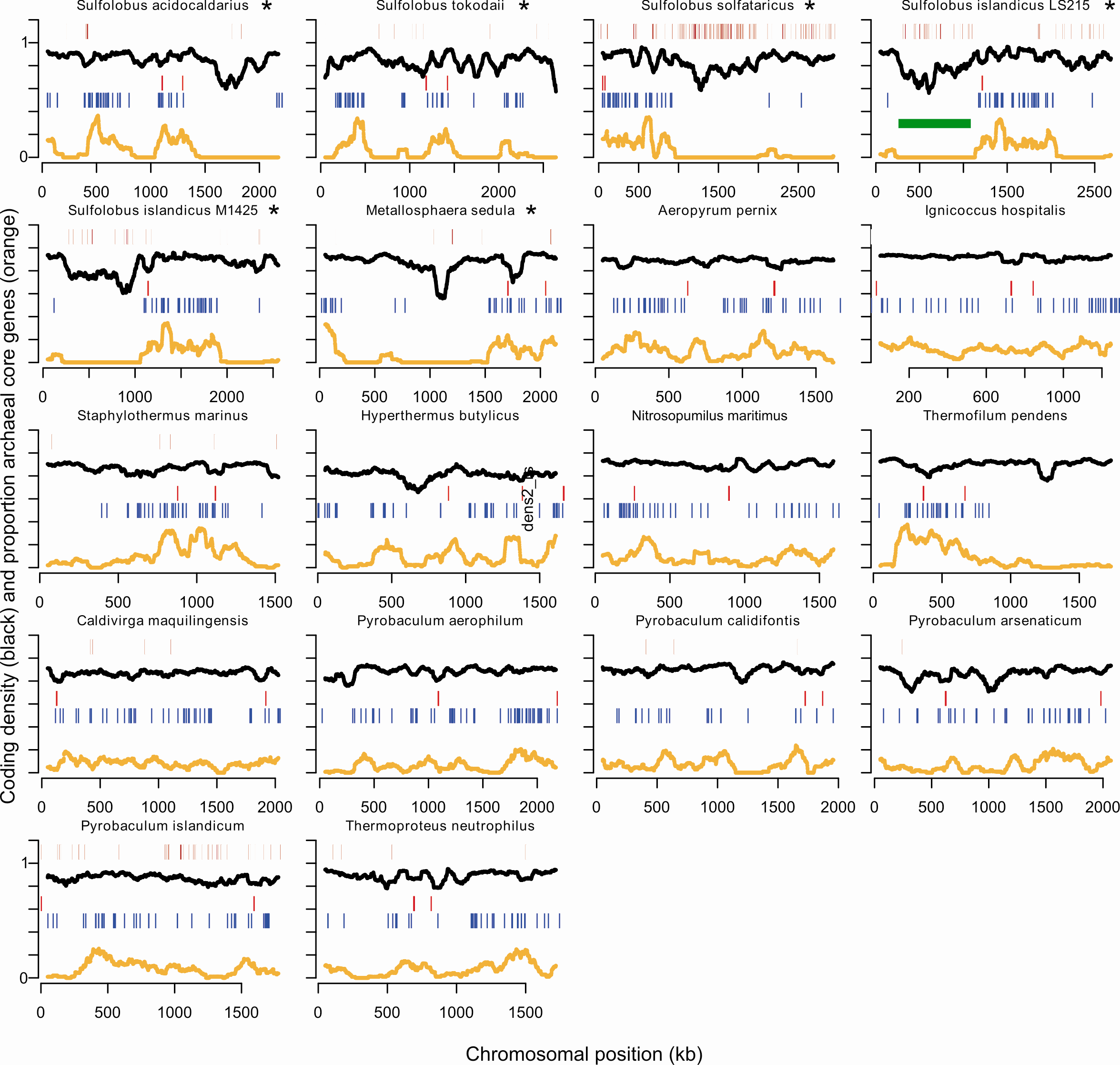


**Supplementary Figure 4.** Distribution of genomic features in completed crenarchaeal genomes. From top to bottom: Brown vertical bars indicate positions of transposons/ transposases (genes with “transposon” or “transposase” in the NCBI gene product description). Black curves indicate protein coding density (in sliding windows of 100 kb transposed in 1 kb steps). Red and blue vertical bars indicate positions of rRNA and tRNA genes, respectively. Yellow curves indicate proportion of archaeal core genes (in sliding windows of 100 kb transposed in 1 kb steps). The green horisontal bar in *S. islandicus* LS215 indicates a region enriched in gene insertions and deletions (Re*no et a*l. 2009). Species labeled with an asterisk belong to the order Sulfolobales.


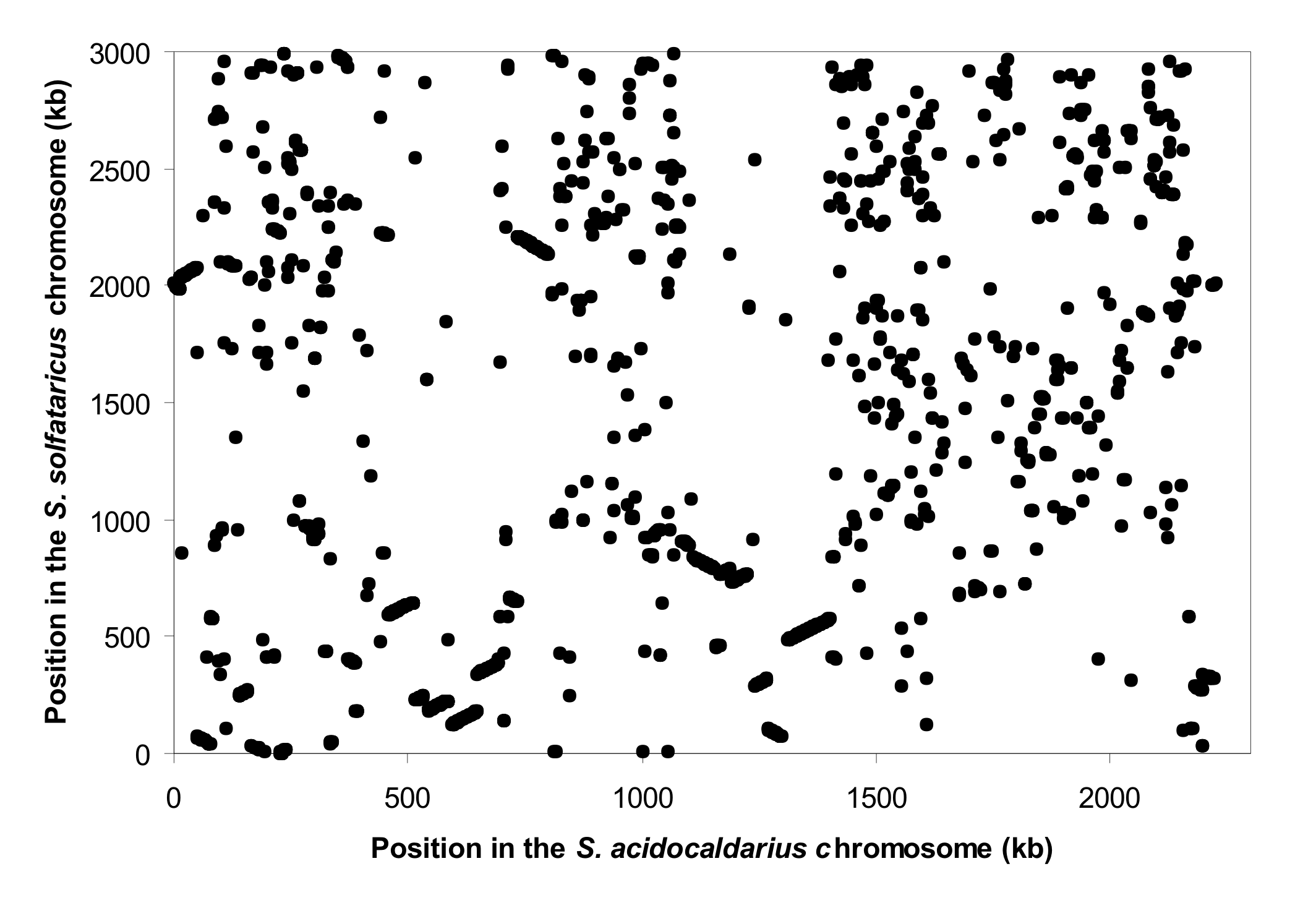


**Supplementary Figure 5.** Chromosome positions for *S. acidocaldarius* and *S. solfataricus* orthologous genes.


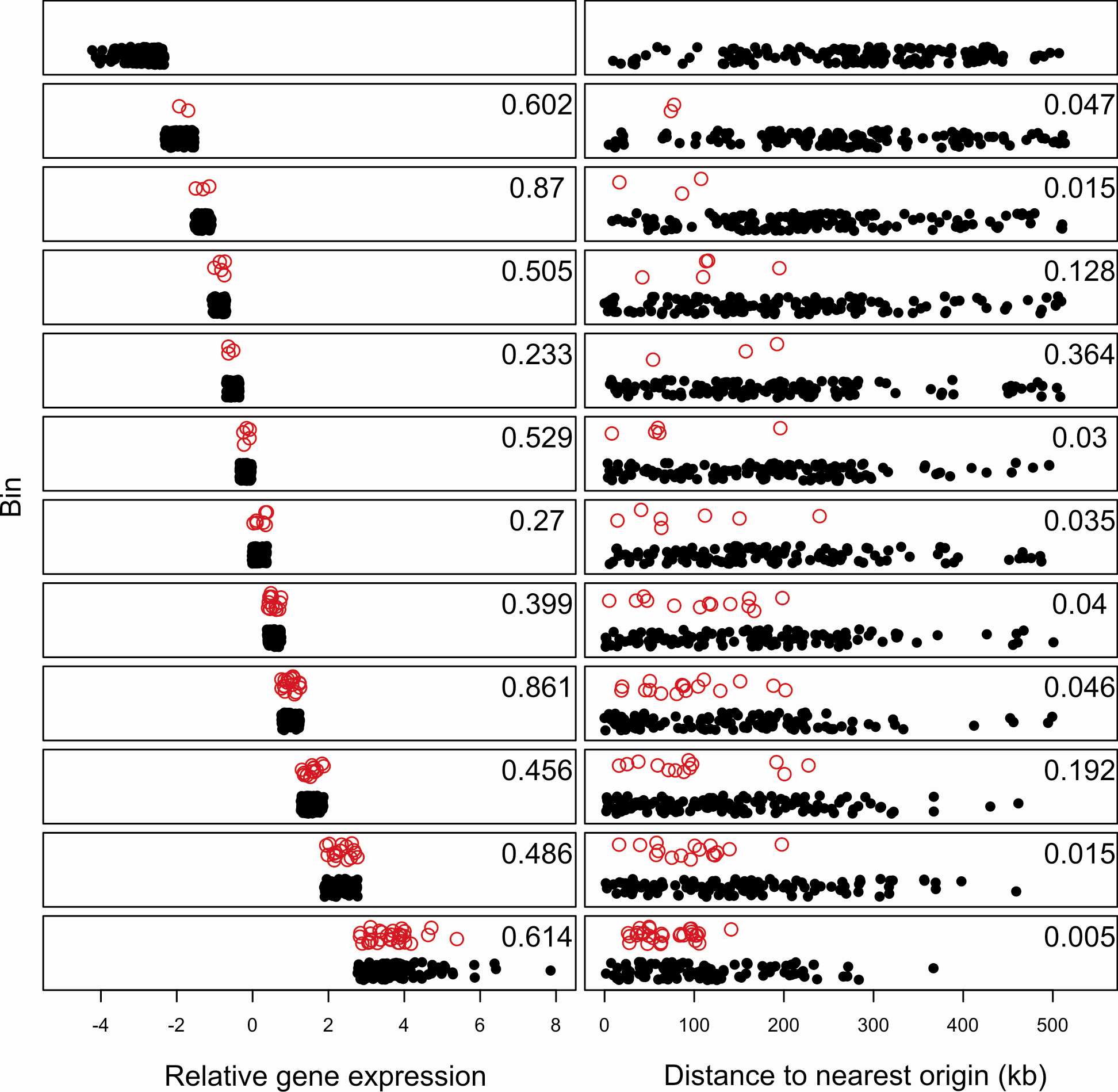


**Supplementary Figure 6.** Gene expression (left column) and distance to nearest origin (right column) within groups of genes binned according to expression level in exponential phase *S. acidocaldarius* cultures. Open (red) and filled (black) circles represent archaeal core and non-core genes, respectively (the bin with lowest expression values includes non-core genes only). Numbers indicate *P*-values from Mann-Whitney tests comparing the distribution of gene expression (left) and distance to nearest origin (right) between the two groups in the different bins.
